# Considerable knock-on displacement of metal atoms under a low energy electron beam

**DOI:** 10.1038/s41598-017-00251-3

**Published:** 2017-03-15

**Authors:** Hengfei Gu, Geping Li, Chengze Liu, Fusen Yuan, Fuzhou Han, Lifeng Zhang, Songquan Wu

**Affiliations:** 1 Institute of Metal Research, Chinese Academy of Sciences, 72 Wenhua Road, Shenyang, 110016 People’s Republic of China; 2 University of Chinese Academy of Sciences, 19 Yuquan Road, Beijing, 100049 People’s Republic of China; 3 University of Science and Technology of China, 96 Jinzhai Road, Hefei, 230026 People’s Republic of China; 4 Key Laboratory of Optoelectronic Materials Chemical and Physics, Fujian Institute of Research on The Structure of Matter, Chinese Academy of Sciences, 155 Yangqiao Road West, Fuzhou, 350002 People’s Republic of China

## Abstract

Under electron beam irradiation, knock-on atomic displacement is commonly thought to occur only when the incident electron energy is above the incident-energy threshold of the material in question. However, we report that when exposed to intense electrons at room temperature at a low incident energy of 30 keV, which is far below the theoretically predicted incident-energy threshold of zirconium, Zircaloy-4 (Zr-1.50Sn-0.25Fe-0.15Cr (wt.%)) surfaces can undergo considerable displacement damage. We demonstrate that electron beam irradiation of the bulk Zircaloy-4 surface resulted in a striking radiation effect that nanoscale precipitates within the surface layer gradually emerged and became clearly visible with increasing the irradiation time. Our transmission electron microscope (TEM) observations further reveal that electron beam irradiation of the thin-film Zircaly-4 surface caused the sputtering of surface α-Zr atoms, the nanoscale atomic restructuring in the α-Zr matrix, and the amorphization of precipitates. These results are the first direct evidences suggesting that displacement of metal atoms can be induced by a low incident electron energy below threshold. The presented way to irradiate may be extended to other materials aiming at producing appealing properties for applications in fields of nanotechnology, surface technology, and others.

## Introduction

Interactions of high-energy particles such as electrons, neutrons, protons, and ions with crystalline lattices of materials give rise to defects such as vacancies, interstitials, electron excitation, ionization, and so on^1–7^. The point defects that survive vacancy-interstitial recombination and sink (dislocations, grain and phase boundaries, etc.) absorption may aggregate, leading to defect clusters including dislocation loops, voids, and localized compositional changes^1, 2^. These irradiation-induced microscopic defects and defect clusters are referred to as radiation damage, which results in changes in physical, chemical and mechanical properties of materials, in aggregate causing macroscopically observable degradation effects such as void swelling, embrittlement, irradiation-induced hardening, growth, and creep, and others, known as radiation effects^2^. Among energetic particles mentioned above, neutrons, as well as protons and ions, can cause considerable radiation damage on bulk crystalline solids and then result in undesirable radiation effects. One example is the degradation of components of nuclear reactors as a result of fast neutron (>200 keV) irradiation^2, 8^. On the contrary, electron irradiation is usually thought to have an insignificant effect on the bulk crystalline system. Only radiation damage due to fast electron (>10 keV) irradiation can draw attention when observation and structural characterization of materials are carried out in a TEM at a high electron accelerating voltage (the voltage of electron beam in TEMs ranges typically between 100 and 300 kV and in a few high-voltage instruments may exceed 1 MV)^3–5^. Fast electrons interact with the nuclei and the electron system in the target^1–7^. Their damage on inorganic crystalline solids takes two principal forms: knock-on atomic displacement and ionization^3^. The former one occurs via electron-nucleus scattering and is termed “knock-on damage” within a crystalline solid or “sputtering” if it occurs on solid surface^4^. For metals, the primary damage way is by knock-on atomic displacement^4^. Due to momentum conversation, only a tiny fraction of the impinging electron energy can be transferred to a nucleus, so a rather high electron energy (an incident-energy threshold) is required to displace a lattice atom from its original position, although the binding energy of lattice atoms is very small (~5–60 eV) compared to the impinging fast electron energy^1–7^. In order to avoid knock-on atomic displacement, it is commonly thought that the only sure way is to use an incident electron energy below the incident-energy threshold of the target material^4, 5^.

With the aim of studying how electron beam irradiation changes the structures and properties of thin film solids or nanosystems, an increasing number of irradiation experiments were carried out in a TEM using a high energy electron beam. In general, radiation damage of electron beam is undesirable, however, recent experiments have demonstrated that it can have beneficial effects^6, 7, 9–12^. Examples are precise cutting of single-walled carbon nanotubes using electron beam^13^, electron-beam-assisted coalescence or joining of single-walled carbon nanotubes^14–16^ and metallic nanowires^17^, interesting phenomena due to electron beam irradiation such as phase transformation in graphite^18, 19^, α-FeSi_2_^20^, and Sn-based nanowires^21^, controlled growth-reversal of catalytic carbon nanotubes^22^, and extreme pressure inside carbon nanotubes^23, 24^, electron-beam-induced formation of nanostructures like carbon onions^25^, double-walled nanotubes^26^, nanopores^27^, alumina nanocapsules^28^, silicon nanocrystals^29, 30^, and crystalline aluminum borate nanowires^31^, restructuring of NaREF_4_ nanocrystals under electron beam irradiation^32^, just to mention a few. In these examples, the applied electron energies are all equal to or greater than 200 keV and even up to MeV, which makes possible the atomic displacement. However, to date research of electron irradiation of inorganic solids with a low energy (<100 keV) electron beam is still limited^33–39^ and the reported studies mainly focus on graphene^33–37^. Also, our literature survey has reflected that there has been no attempt to investigate radiation damage or radiation effect of low energy electron beam on metals.

In this study, we irradiated surfaces of recrystallized α-type Zircaloy-4 (Zr-1.50Sn-0.25Fe-0.15Cr (wt.%)) at room temperature using stationary electron beam with a small diameter at 30 kV accelerating voltage in a FEI Inspect F50 field-emission scanning electron microscope (FE-SEM). It was striking to find that under irradiation nanoscale precipitates within the surface layer of bulk Zircaloy-4 gradually emerged and became clearly visible with increasing the irradiation time. Furthermore, TEM investigations using a combination of bright field (BF) TEM imaging, selected area electron diffraction (SAED), fast Fourier transformation (FFT) diffraction, and inverse fast Fourier transformation (IFFT) imaging reveal that under irradiation with 30 keV electrons the displacement of zirconium atoms at the surface of thin-film Zircaloy-4 indeed occurred, exhibiting in the forms of sputtering of surface α-Zr atoms, nanoscale atomic reconstructions in the α-Zr matrix and disorder formation in precipitates. These results are beyond the common expectation as the incident electron energy under study is much lower than the theoretically predicted incident-energy threshold of zirconium for knock-on atomic displacement and we attribute them to a considerably high specimen current density and a relatively high energy deposition rate in the specimens.

## Results and Discussion

**Figure 1:**
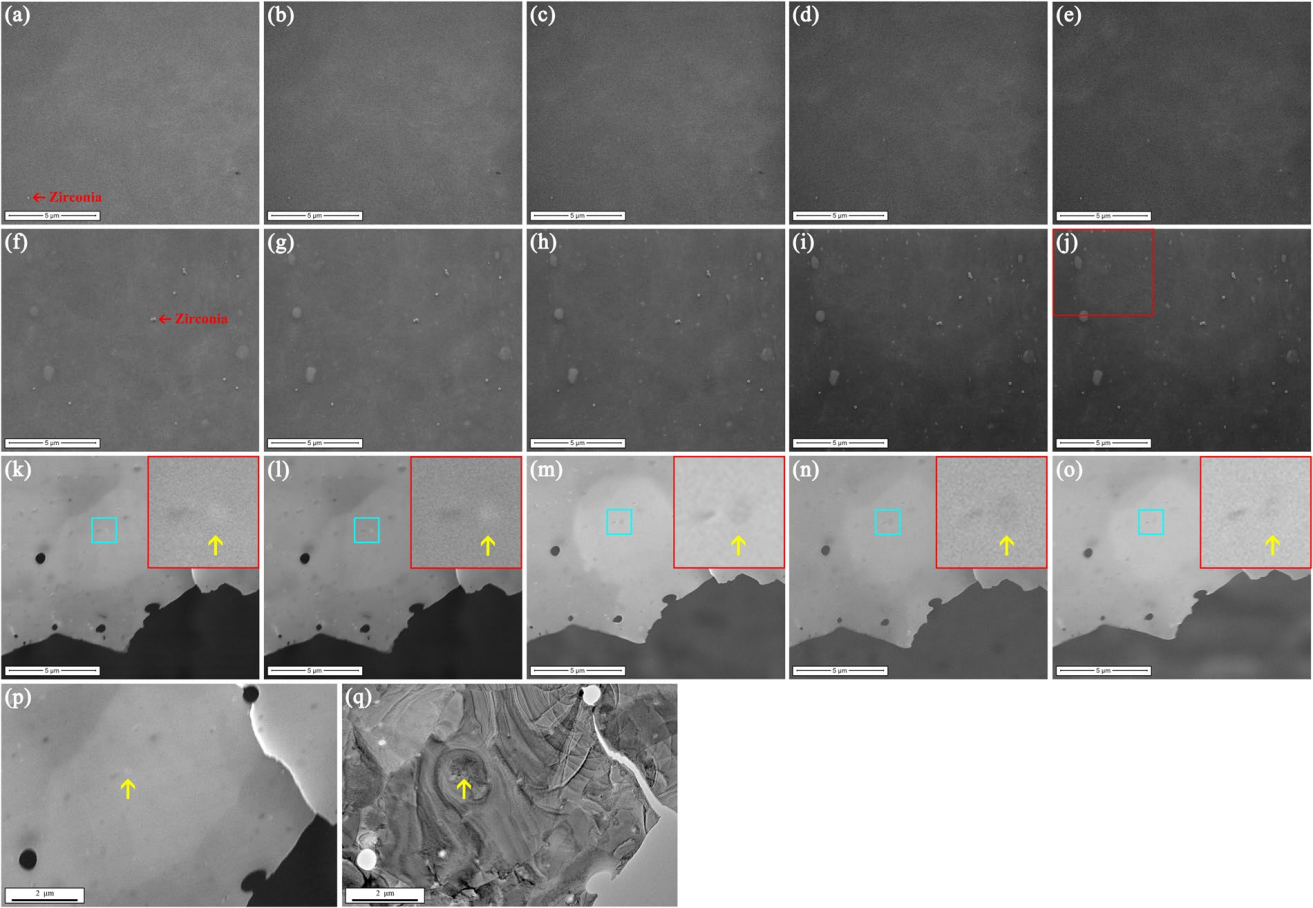
Surface morphology evolutions of pure Zr and Zircaloy-4 specimens under irradiation at room temperature with focused and stationary electron beam at an incident energy of 30 keV in the FE-SEM. (**a–e**) SEM images of the selected surface area of the polished bulk pure zirconium specimen after irradiation for 1, 8, 16, 24, and 32 electron beam scans, respectively. (**f**–**j**) SEM images of the selected surface area of the polished bulk Zircaloy-4 specimen after irradiation for 1, 8, 16, 24, and 32 electron beam scans, respectively. The clearly visible ball-shaped zirconia particles (red arrows in Fig. 1a and f) suspended on the surface indicate that the SEM images were taken when the surface was clearly focused. (**k**–**o**) SEM images of the selected thin film region in the vicinity of the hole of the Zircaloy-4 TEM specimen after irradiation for 1, 32, 64, 96, and 128 electron beam scans, respectively. The area outlined by a blue square in every image is expanded in its corresponding inset outlined by a red square, which shows the morphology of the precipitate of interest (yellow arrow in the inset). (**p**,**q**) SEM and TEM images showing that the electron beam irradiation in the FE-SEM and the microstructure observation in the TEM were performed on the same thin film region of the Zircaloy-4 TEM specimen containing the precipitate of interest (yellow arrows in Fig. 1p and q).

**Figure 2:**
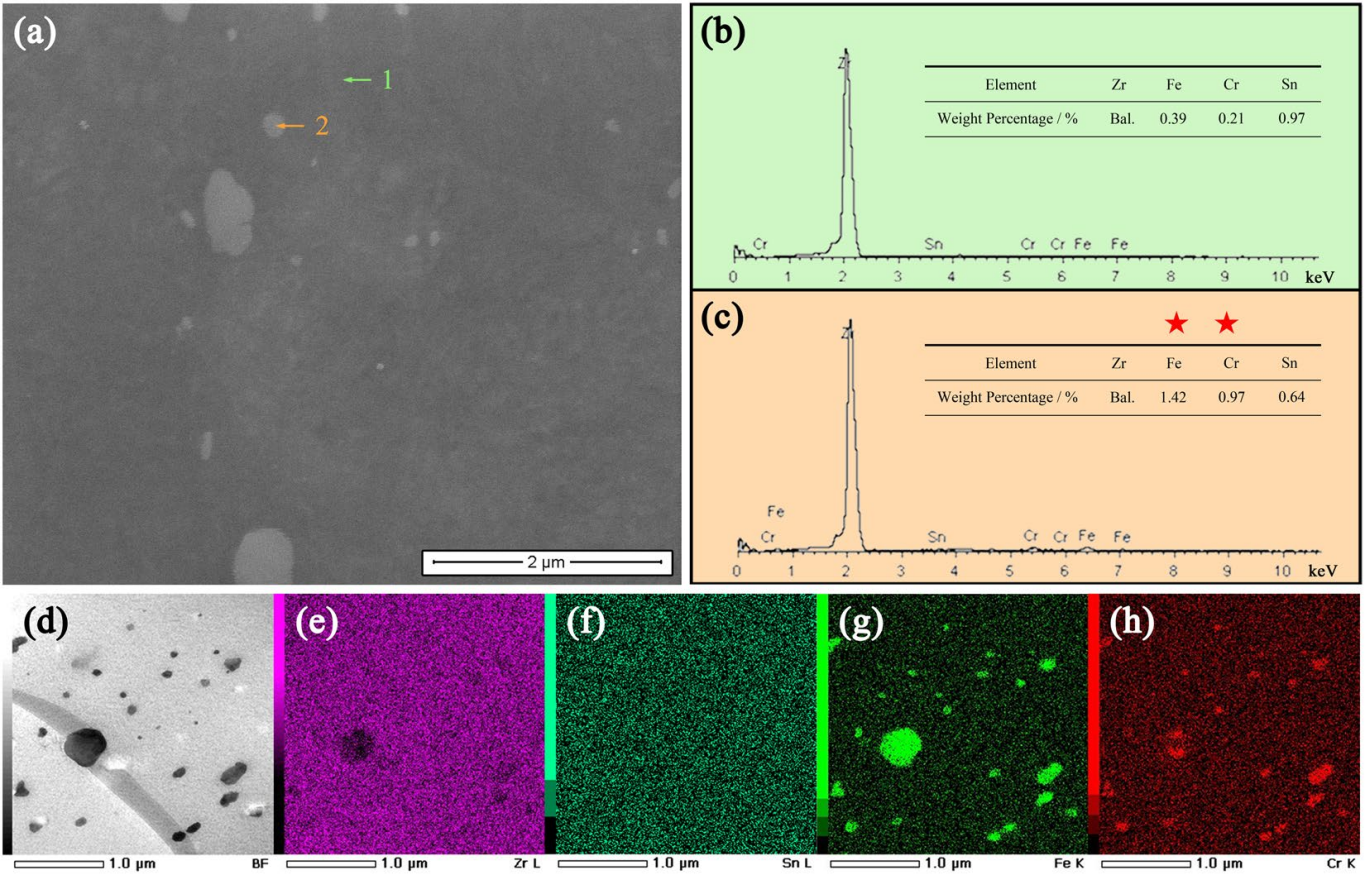
Compositional analysis. (**a**) The expanded surface morphology of the area outlined by a red square in Fig. 1j. (**b**–**c**) EDS spectra and compositions corresponding to Point 1 and 2 in Fig. 2a, respectively. (**d–h**) STEM image showing morphologies of precipitates (ball-shaped nanoparticles with dark contrast) in Zircaloy-4 (**d**) and its corresponding Zr L (**e**), Sn L (**f**), Fe K (**g**), and Cr K (**h**) elemental maps.

We begin by preparing bulk specimens (5 mm in thickness) of recrystallized pure Zr (>99.9 wt.%) and Zricaloy-4 (Zr-1.50Sn-0.25Fe-0.15Cr (wt.%)) with α-Zr phase of P6_3_/mmc space group^40^, and polishing their surfaces (see Methods for detail). On these two polished surfaces irradiation with focused electron beam at a 30 kV accelerating voltage in the FE-SEM was performed at room temperature for 32 electron beam scans (imaging was simultaneously carried out and every scan lasts 35 s to obtain a SEM image), respectively (see Methods for detail). Their SEM morphological evolutions under irradiation are shown in Fig. 1a–e and f–j, respectively. By comparison, it can be found that as irradiation continued up to 32 scans, an increasing number of ball-shaped nanoparticles with bright contrast and various diameters (~35–500 nm) gradually emerged on the Zircaloy-4 surface and their profiles became clearly visible (see Supplementary video), whereas the surface of pure Zr remained unchanged. These nanoparticles should be assigned to the precipitates in Zircaloy-4, as nanoscale precipitates resulting from addition of alloying elements exist in Zircaloy-4 rather than in pure Zr. To further confirm this assignment, compositional analysis was carried out. Energy dispersive X-ray spectrum (EDS) results (Fig. 2a–c) reveal that the newly presented nanoparticles (Point 2 in Fig. 2a) after irradiation on the zircaloy-4 surface possess a higher content of alloying elements Fe and Cr compared with the matrix of Zircaloy-4 (Point 1 in Fig. 2a). It is in line with the fact that the precipitates in Zircaloy-4 (ball-shaped nanoparticles with dark contrast in Fig. 2d) ~30–450 nm in diameter are rich in Fe and Cr (Fig. 2g,h). Thus, it can be concluded that performing focused and stationary electron beam at a low incident energy of 30 keV in the FE-SEM enables the precipitates in Zircaloy-4 to emerge on its surface with clear profiles. This phenomenon can be interpreted as a radiation effect, as it is a macroscopically observable change of surface morphology due to electron beam irradiation. We noticed that this radiation effect can be clearly observed only when the diameter of electron beam in the FE-SEM is small enough like our experimental condition (the magnification should be at 20,000× or larger and the irradiation area is ~14.9 × 12.8 μm^2^ or smaller, correspondingly).

To investigate the atomic-scale radiation damage of electron beam on the precipitates as well as the α-Zr matrix, a TEM specimen of Zircaloy-4 was prepared (see Methods for detail), and then in this specimen a thin film region containing a precipitate of interest (yellow arrows in Fig. 1k–q) was selected to be irradiated at room temperature for 32 scans in the FE-SEM (like the case for the bulk Zircaloy-4 surface) and subsequently observed in the TEM. Such alternative treatments of electron beam irradiation in the FE-SEM and microstructure observation in the TEM were repeated for 4 times (TEM observations were carried out after irradiation for 32, 64, 96, and 128 scans, respectively).

**Figure 3:**
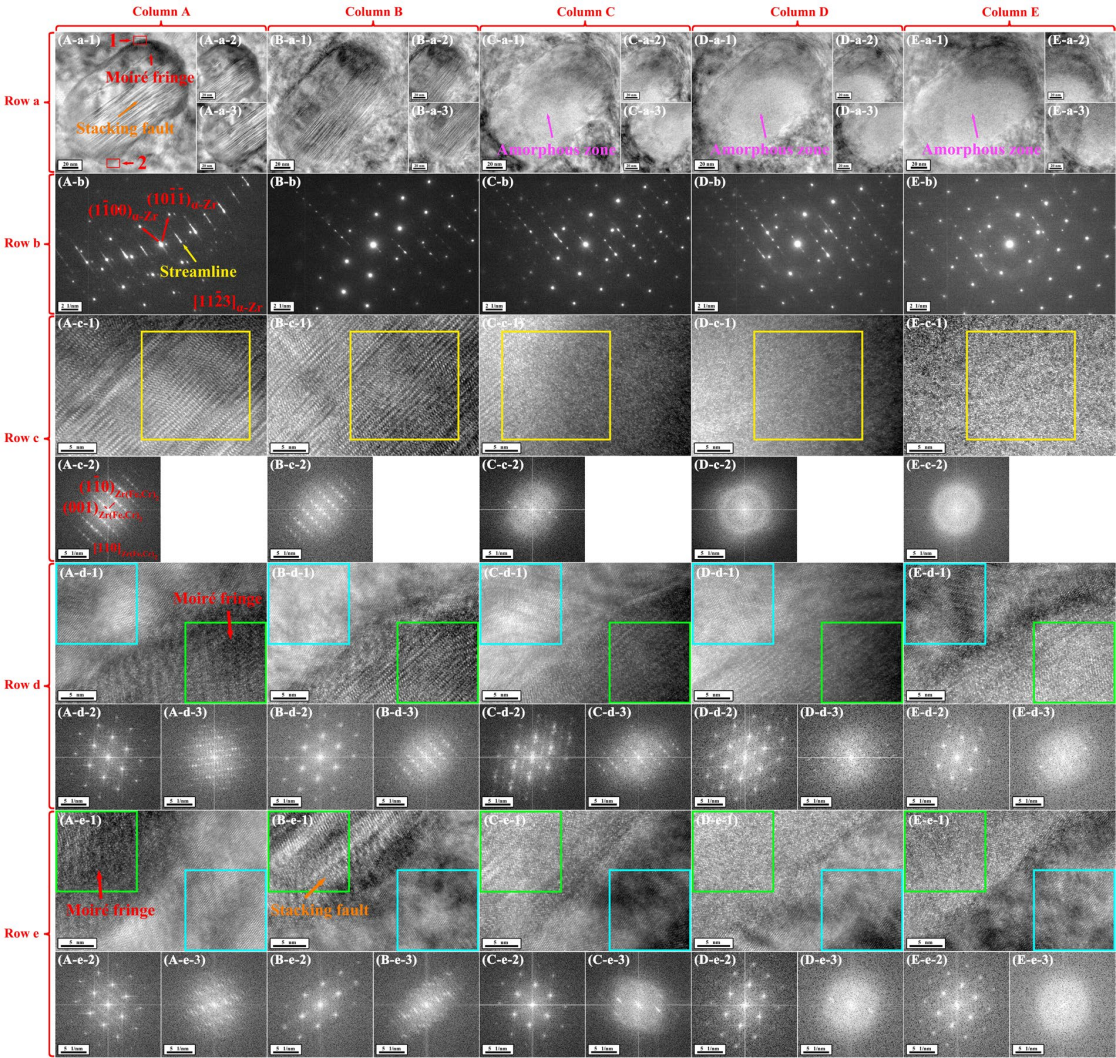
TEM observations of the precipitate of interest and its surrounding α-Zr matrix before and after irradiation at room temperature with focused and stationary electron beam at an incident energy of 30 keV in the FE-SEM viewed along the [11$\bar{2}$3]_α-Zr_ direction. Column A–E of Fig. 3 shows TEM results before and after irradiation for 32, 64, 96, and 128 electron beam scans, respectively. Row a of Fig. 3 shows BF TEM images of the precipitate of interest and its surrounding α-Zr matrix. Row b of Fig. 3 shows composite SAED patterns of the precipitate of interest and its surrounding α-Zr matrix. Row c–e of Fig. 3 show HRTEM images and their corresponding FFT diffraction patterns corresponding to the center (not the same position), the up-left corner (Area 1 in Fig. 3(A-a-1)) and the down-right corner (Area 2 in Fig. 3(A-a-1)) of the precipitate of interest, respectively. In Row c of Fig. 3, the FFT diffraction pattern in every column corresponds to the area outlined by a yellow square in the corresponding HRTEM image. In Row d and e of Fig. 3, the left and right FFT diffraction patterns in every column of Fig. 3 correspond to the areas outlined by blue and green squares in the corresponding HRTEM image, respectively.

Prior to irradiation, detailed pre-observations of the selected precipitate were carried out in the TEM. Figure 3(A-a-1)–(A-a-3) show the BF TEM morphology of the precipitate before irradiation viewed along the [11$\bar{2}$3]_α-Zr_ direction. The FFT image (Fig. 3(A-c-2)) corresponding to the central area of the precipitate (Fig. 3(A-c-1)) shows a clear periodic FFT diffraction pattern. Through indexing, it can be identified that the precipitate is Zr(Fe,Cr)_2_ phase of P6_3_/mmc space group^41^ and the zone axis in Fig. 3(A-c-2) is along the [110]_Zr(Fe,Cr)2_ direction. Thus, the orientation relationship between the matrix (α-Zr phase) and the precipitate (Zr(Fe,Cr)_2_ phase) here can be written as:

$${[11\bar 23]_{{\rm{\alpha }} - {\rm{Zr}}}}||{[110]_{{\rm{Zr}}{{\left({{\rm{Fe,Cr}}} \right)}_2}}}$$

**Figure 4:**
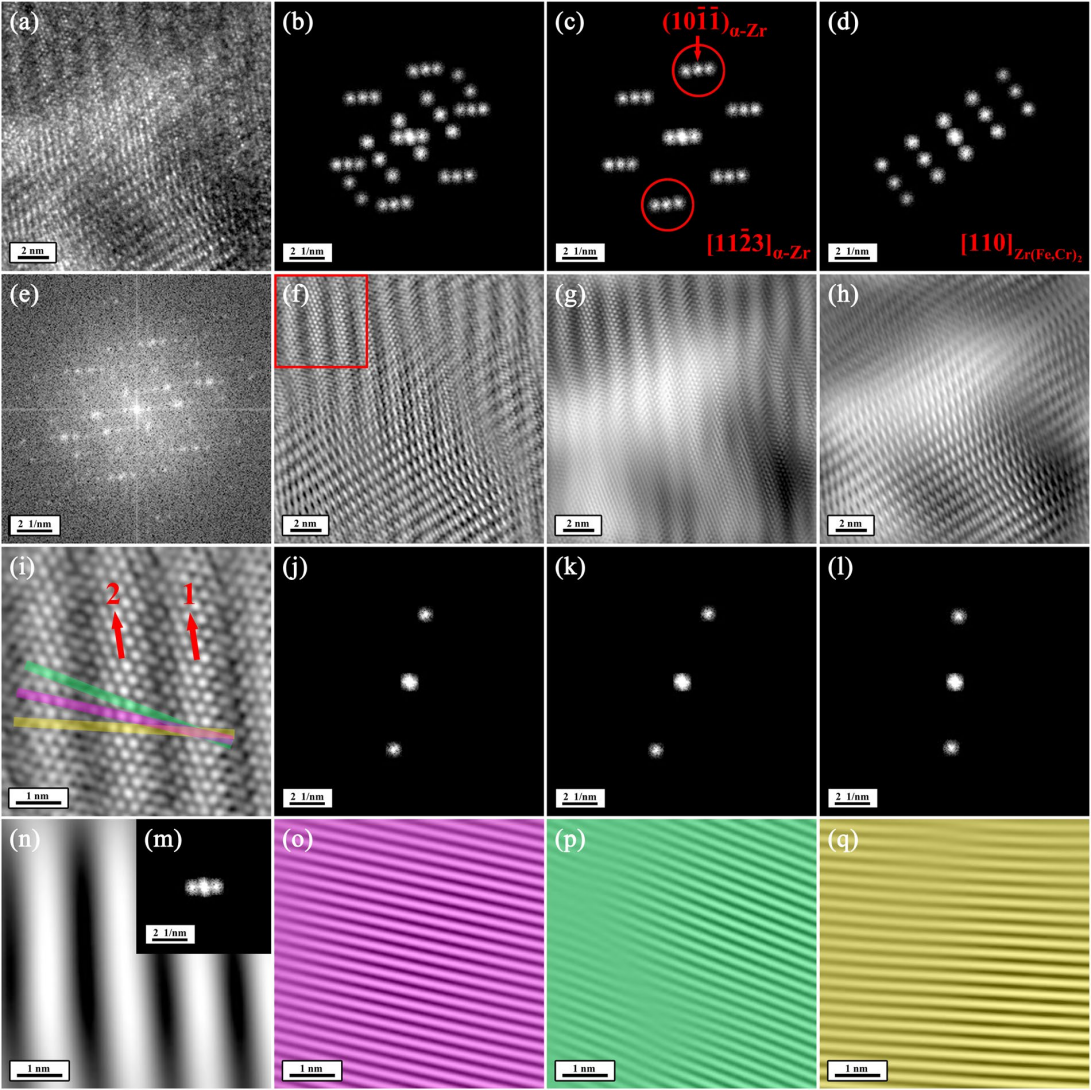
Moiré fringes at the perimeter of the precipitate of interest. (**a**) HRTEM image showing an area where fringes disappeared at the interior of the precipitate viewed along the [11$\bar{2}$3]_α-Zr_ direction. (**e**) The FFT diffraction pattern corresponding to Fig. 4a. (**b**,**c** and **d**) The partially masked FFT diffraction patterns corresponding to Fig. 4c. (**f,g** and **h**) The noise-filtered IFFT images corresponding to Fig. 4a obtained by using the partially masked FFT diffraction patterns in Fig. 4(b,c and d), respectively. In Fig. e, b and c, it can be seen that every FFT diffraction spot (including the central FFT diffraction spot) belonging to α-Zr phase along [11$\bar{2}$3]_α-Zr_ diverges into three spots. (**i**) The expanded morphology of the area outlined by a red square in Fig. 4f. (**j**,**k** and **l**) The three diverged FFT diffraction spots corresponding to the (10$\bar{1}\bar{1}$)_α-Zr_ planes, respectively. (**o,p** and **q**) The IFFT images corresponding to Fig. 4i obtained by using the masked FFT diffraction spots in Fig. 4j, k and l, respectively. (**m**) The three diverged spots of the central FFT diffraction spot. (**n**) The IFFT image corresponding to Fig. 4i obtained by using the masked FFT diffraction spots in Fig. 4m. The divergence of the FFT diffraction pattern corresponding to α-Zr phase along [11$\bar{2}$3]_α-Zr_ as well as the central FFT diffraction spot is due to the presence of the fringes. Take the (10$\bar{1}\bar{1}$)_α-Zr_ planes for example. The (10$\bar{1}\bar{1}$)_α-Zr_ planes on fringes (pink bar in Fig. 4i) can still form periodic planes (Fig. 4o). The (10$\bar{1}\bar{1}$)_α-Zr_ planes on Fringe 1 in Fig. 4i can line up with their two adjacent (10$\bar{1}\bar{1}$)_α-Zr_ planes on Fringe 2 in Fig. 4i (green and yellow bars in Fig. 4i) and form periodic planes (Fig. 4p and q), respectively. In addition, the fringes themselves are periodic, resulting in the divergence of the central FFT diffraction spot as seen in Fig. 4n and m.

A close observation indicates that not only stacking faults but also moiré fringes existed on the precipitate as described below. Figure 3(A-a-1) shows that the right half of the precipitate exhibiting lamellar morphology (orange arrow in Fig. 3(A-a-1)). These lamellar contrasts are almost parallel to each other and also parallel to the (001)_Zr(Fe,Cr)2_ planes, but their spacings are not uniform. Seen from the composite SAED pattern of the precipitate and its surrounding matrix (Fig. 3(A-b)), it is apparent that bright streamlines (yellow arrow in Fig. 3(A-b)) existed between the diffraction spots corresponding to the (001)_Zr(Fe,Cr)2_ planes (making undiscernible the [110]_Zr(Fe,Cr)2_ SAED pattern), suggesting that the lamellar contrasts are attributed to stacking faults in the precipitate^42, 43^. Apart from these stacking faults, the perimeter of the precipitate exhibited morphology of parallel fringes with an equal spacing (red arrow in Fig. 3(A-a-1)). These fringes are found to, on one hand, terminate at the interface of the precipitate and the matrix, and, on the other hand, disappear at the interior of the precipitate. To investigate these fringes, a local area where they disappeared at the interior of the precipitate (Fig. 4a) was selected to analyze. The IFFT image (Fig. 4f) corresponding to Fig. 4a, which reduces the noise, shows that parallel fringes can be clearly observed on the up-right part of the image. A detailed look indicates the up-right and down-left parts of the image exhibited the atomic structures of α-Zr phase along [11$\bar{2}$3]_α-Zr_ and Zr(Fe,Cr)_2_ phase along [110]_Zr(Fe,Cr)2_, respectively, as confirmed by their corresponding FFT diffraction patterns (Fig. 4c and d), respectively. These results reveal that the matrix covered on the perimeter of the precipitate and that the fringes only appeared on where the matrix and the precipitate were overlapped. Thus, these fringes can be assigned to moiré fringes, which are formed under electron beam if two crystals are superimposed at a suitable mutual orientation^44^. The moiré fringes together with the stacking faults provide two interesting characteristics of morphology to study radiation damage of electron beam.

Figure 1k–o demonstrate that irradiation with 30 keV electrons in the FE-SEM were performed on the pre-selected thin film region of Zircaloy-4 for 1 (almost before irradiation), 32, 64, 96 and 128 scans, respectively, and their insets show the SEM morphological change of the precipitate of interest during irradiation. It is noticed that the contrast of the precipitate, which was originally bright (the inset in Fig. 1k), turned into dark after irradiation (the insets in Fig. 1m–o). The same was also true for other precipitates as seen in Fig. 1o. The TEM observations of the precipitate after irradiation in the FE-SEM are summarized in Fig. 3. To monitor the structural changes during the irradiation process, three parts of the precipitate including the center (not the same position), the up-left corner (the same position) and the down-right corner (the same position), were selected for HRTEM observation and FFT diffraction pattern analysis as shown in Row c-e of Fig. 3, respectively. After irradiation for 32 scans, a considerable fraction of moiré fringes disappeared (Fig. 3(B-a-1)–(B-a-3)) and what was beneath them can be seen. For example, after irradiation the up-left and down-right corners of the precipitate exhibited the atomic structure of Zr(Fe,Cr)_2_ phase along [110]_Zr(Fe,Cr)2_(Fig. 3(B-d-1)) and the stacking faults (Fig. 3(B-e-1)), respectively. When irradiation was up to 64 scans, no moiré fringes can be observed on the precipitate (Fig. 3(C-a-1)–(C-a-3)). The disappearance of moiré fringes suggests that the atoms of α-Zr matrix (mainly the Zr atoms) that initially covered on the precipitate were removed into vacuum by electron beam irradiation. This, in return, can explain the appearance of precipitates on the bulk Zircaloy-4 surface under irradiation. Also, after irradiation for 64 scans, a considerable fraction of stacking faults disappeared (Fig. 3(C-a-1)–(C-a-3)), leading to the weaker streamline contrasts and the discernible [110]_Zr(Fe,Cr)2_ diffraction spots of the precipitate in the composite SAED pattern (Fig. 3(C-b)). As irradiation continued up to 128 scans, almost no stacking faults can be seen (Fig. 3(E-a-1)–(E-a-3)). Moreover, after irradiation for 64 scans, a bright zone was present inside the precipitate (pink arrow in (Fig. 3(C-a-1)). The HRTEM image (Fig. 3(C-c-1)) indicates that the atomic structure in this zone exhibited a random distribution of atoms, resulting in broad halo rings in its corresponding FFT diffraction pattern (Fig. 3(C-c-2)). These HRTEM and FFT results reveal that an amorphous structure was formed inside the newly presented zone (the SEM contrast change of the precipitate during irradiation should be attributed to the formation of the amorphous zone). Seen from Fig. 3(C-a-1), (D-a-1) and (E-a-1), the longer the irradiation time, the lager the size of the amorphous zone became. As confirmed in the composite SAED patterns (Fig. 3(C-b), (D-b) and (E-b)), broad halo rings started to appear and their contracts increased in intensity with increasing the irradiation time. For details, HRTEM images and FFT diffraction patterns in Row d and e of Fig. 3 show how the atomic structure of the precipitate and the stacking faults were gradually restructured by irradiation, leading to forming an amorphous structure, respectively. The disappearance of the stacking faults together with the formation of the amorphous zone suggest that the atoms of the precipitate (including the Zr, Sn, Fe, Cr atoms) were displaced to a large extend and probably sputtered into vacuum by electron beam irradiation.

**Figure 5:**
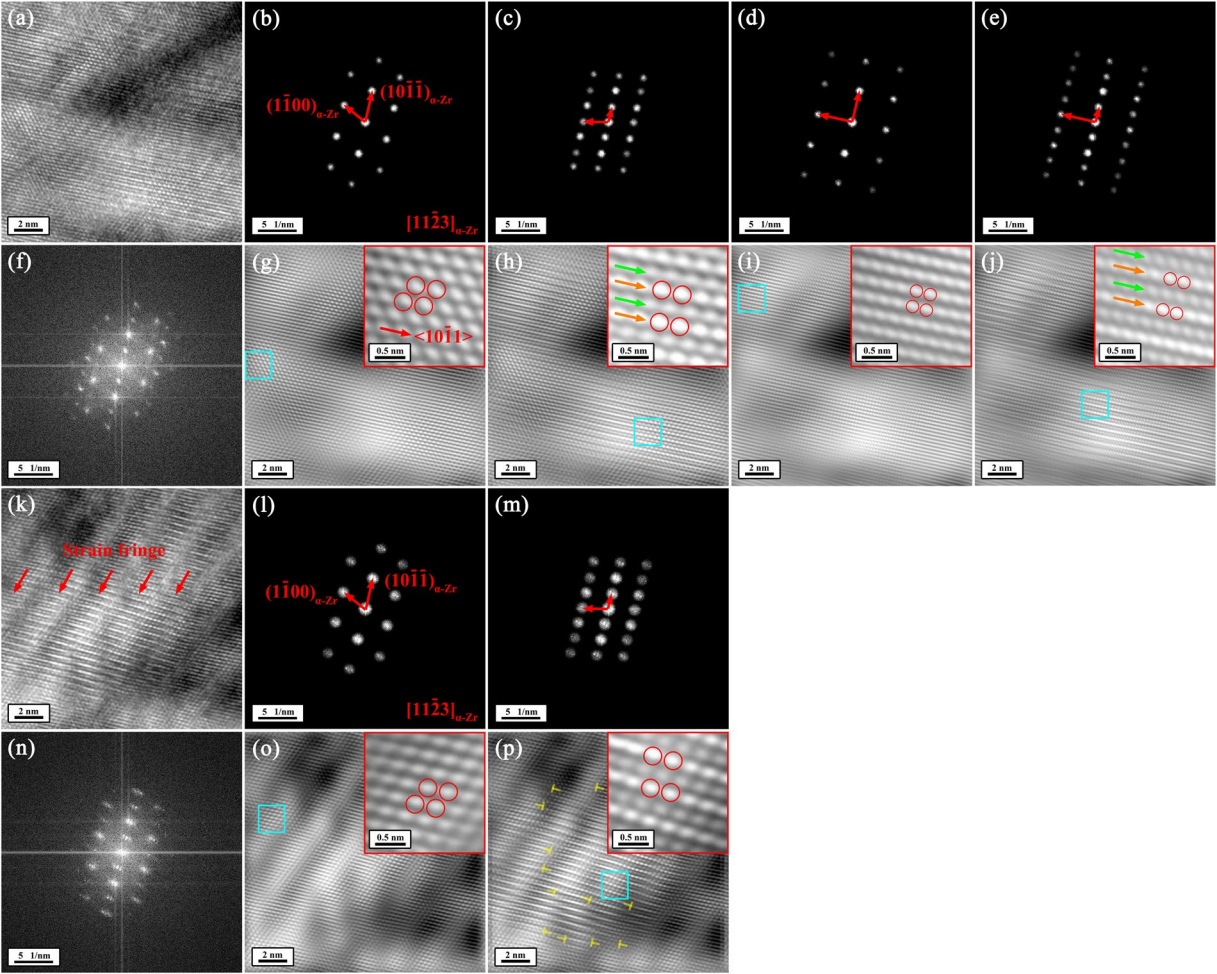
Changes of atomic structure within the α-Zr matrix after irradiation at room temperature with focused and stationary electron beam at an incident energy of 30 keV in the FE-SEM viewed along the [11$\bar{2}$3]_α-Zr_ direction. (**a**) and (**k**) HRTEM images of two typical areas of the α-Zr matrix containing electron-beam-induced changes of atomic structure. (**f**) and (**n**) The FFT diffraction patterns corresponding to Fig. 5a and k, respectively. (**b**,**c**,**d** and **e**) The partially masked FFT diffraction patterns corresponding to Fig. 5f. (**g**,**h**,**i** and **j**) The noise-filtered IFFT images corresponding to Fig. 5a obtained by using the partially masked FFT diffraction patterns in Fig. 5b,c,d and e, respectively. The insets in Fig. 5g,h,i and j show the expanded morphologies of the areas outlined by blue squares in Fig. 5g,h,i and j, respectively. (**l**) and (**m**) The partially masked FFT diffraction patterns corresponding to Fig. 5n. (**o**) and (**p**) The noise-filtered IFFT images corresponding to Fig. 5k obtained by using the partially masked FFT diffraction patterns in Fig. 5l and m, respectively. The insets in Fig. 5o and p show the expanded morphologies of the areas outlined by blue squares in Fig. 5o and p, respectively. The yellow ‘T's in Fig. 5p indicate the electron-beam-induced dislocations.

The electron-beam-induced radiation damage occurred not only on the precipitate but also on its surrounding matrix, which have some typical manifestations of atomic structure change as shown in Fig. 5a and k. Seen from Fig. 5a, in spite of the remaining [11$\bar{2}$3]_α-Zr_ atomic structure (the insert of Fig. 5g), localized areas exhibited a new atomic structure (the insert of Fig. 5i), corresponding to a new FFT diffraction pattern (Fig. 5d). Its formation is geometrically due to <10$\bar{1}$1> displacement (red arrow in the inset of Fig. 5g) of atoms within the (10$\bar{1}\bar{1}$)_α-Zr_ planes. Apart from this new atomic structure, another irradiation-induced atomic feature in Fig. 5a is that every two (10$\bar{1}\bar{1}$)_α-Zr_ planes exhibited the same stronger (orange arrows in the inserts of Fig. 5h and j) or weaker (green arrows in the inserts of Fig. 5h and j) contrasts, forming a sort of planar fault. It is like a sinusoidal vibration of (10$\bar{1}\bar{1}$)_α-Zr_ planes along the [11$\bar{2}$3]_α-Zr_ direction. These planar faults result in new periodic atomic structures (red circles in the inserts of Fig. 5h and j), corresponding to new FFT diffraction patterns (Fig. 5c and e). Such an atomic feature can also be seen in Fig. 5k, which is more apparent. In Fig. 5k, moreover, strain fringes can be observed (red arrows in Fig. 5k), among which dislocations are visible (yellow ‘T's in Fig. 5p). The appearance of strain fringes is more likely because of irradiation-induced stress relaxation at the interface of the precipitate and the matrix. The formation of new atomic structures along with the appearance of strain fringes suggest that within the matrix surrounding the precipitate atomic displacement took place upon electron beam irradiation.

Above experiment results have confirmed the occurrence of knock-on displacement of zirconium atoms under electron beam at a low incident energy of 30 keV. Theoretically, a simple expression given by Hobbs for the displacement energy (E_d_) allows us to determine the incident-energy threshold (E_t_) for displacement of atoms of atomic weight A within a lattice network^3, 4^

1 $${{\rm{E}}}_{{\rm{t}}}=\frac{{\left(\frac{100+{\rm{A}}{{\rm{E}}}_{{\rm{d}}}}{5}\right)}^{\frac{1}{2}}-10}{20}$$

where E_t_ is in MeV and E_d_ is in eV. For zirconium, which has an atomic weight of 91.22 ^40^, the minimum energy for atomic displacement within its crystal is ~18 eV^2^, so accordingly the incident-energy threshold for knock-on damage calculated according to Hobbs’ expression is ~433 keV. In the case of sputtering, Hobbs’ expression remains valid, but the energy for displacing a surface atom is much lower because there are fewer other atoms to disrupt. In general, the incident-energy threshold for sputtering is ~50% less than that for knock-on damage^4^. As for zirconium, it was reported to be ~210 keV^2^. By comparison, it is evident that the 30 keV incident electron energy that caused knock-on displacement of zirconium atoms in our experiments is far below the incident-energy threshold of zirconium for knock-on damage (~433 keV) and sputtering (~210 keV). Such a finding leads to our reconsidering of the criteria for knock-on atomic displacement to occur under electron irradiation. Literature indicates that calculating the incident-energy threshold according to Hobbs’ expression assumes that a nucleus is only exposed to one electron, so this assumption is suitable for the situation when the specimen current density is very low, in which a nucleus interacts with one electron at the same time. In this case, the incident electron energy should be the dominate factor that influences atomic displacement and displacement damage cannot occur when the incident electron energy below threshold. This is in line with our TEM observation experience that under electron beam in the TEM, which was maintained at a very low specimen current density of ~40 pA/cm^2^ at a high accelerating voltage of 200 kV, the Zircaloy-4 specimen remained stable during our experimental time (in the scale of hours). However, in the case for the FE-SEM a high specimen current density at the level of mA/cm^2^ and even A/cm^2^ can result from electron probes of very small diameter^5^, like the case for irradiation in our experiments. As our SEM observation indicates that the displacement of zirconium atoms under electron beam at a low accelerating voltage of 30 kV can take place significantly only when the diameter of electron beam in the FE-SEM is small enough, that is only when the specimen current density in the FE-SEM is large enough. Thus, a high specimen current density should mainly contribute to considerable knock-on displacement of zirconium atoms under a low energy electron beam in the FE-SEM. Under such electron beam at a high specimen current density (even though the incident electron energy is very low), a nucleus is likely to simultaneously undergo elastic collisions with multiple electrons and the small momentum that it acquires through the impact of every electron can be accumulated to a large one, making possible its displacement. This might be one explanation, which still needs to be further confirmed. Moreover, electron energy deposition rate increases with decreasing the incident electron energy^3^, so it is higher under electron beam in the FE-SEM compared with that in the TEM, which also possibly contributes to the radiation damage on the Zircaloy-4 surfaces under irradiation in the FE-SEM. The other possible damage mechanism is through heating induced by electron beam irradiation. A higher specimen current density results in a larger increase of specimen temperature^5^, however, the temperature rise is insignificant for metals^4, 5^, in particular for the bulk, in which heat flow is radial in three dimensions^5^, so the irradiation-induced thermal effect cannot be the dominant factor that accounts for atomic displacement. Thus, we believe that a considerably high specimen current density together with a relatively high energy deposition rate are the main reasons for the knock-on displacement of zirconium atoms under focused and stationary electron beam at a low incident energy in the FE-SEM.

## Conclusion

We have demonstrated that, on one hand, irradiation of the bulk Zircaloy-4 under focused and stationary electron beam at an incident energy of 30 keV in the FE-SEM resulted in a striking radiation effect that nanoscale precipitates within the surface layer gradually emerged and became clearly visible with increasing the irradiation time, which can be assigned to the sputtering of surface α-Zr atoms. This phenomenon provides a convenient way to examine size and geometry distribution of precipitates in zirconium alloys. On the other hand, the same electron beam irradiation of the thin-film Zircaloy-4 caused the sputtering of surface α-Zr atoms, the nanoscale atomic restructuring in the α-Zr matrix, and the amorphization of precipitates. These results are the first direct evidences suggesting that displacement of metal atoms can be induced by a low incident electron energy below threshold, which we explain as resulting from a combination of a considerably high specimen current density of focused electron beam and a relatively high energy deposition rate owing to a low incident electron energy. The radiation damage of electrons on the Zircaloy-4 surfaces is so significant that may draw attention on surface degradation of zirconium alloys due to electron irradiation (such as β rays) under practical conditions in nuclear reactors. In addition, as restructuring of the α-Zr matrix can occur at the nanoscale under a low energy electron beam, irradiation with such an electron beam may be a viable route to study the gradual process of phase transformation within inorganic solids. Moreover, irradiation of other materials with electron beam at a low incident energy at a high specimen current density in a FE-SEM may similarly cause considerable radiation damage and perhaps lead to promising physical and chemical properties for applications in a variety of fields, such as nanotechnology, surface technology, and others. With the help of a much larger effective irradiation area in a FE-SEM compared to that in a TEM, their applicability in the practical uses is more likely to be achieved.

## Methods

### Materials

Recrystallized pure Zr (>99.9 wt.%) and Zricaloy-4 (Zr-1.50Sn-0.25Fe-0.15Cr (wt.%)) with α-Zr phase of P6_3_/mmc space group^40^ were prepared. Both of them were cut into bulk specimens 5 mm in thickness by electrical discharge machining and their surfaces were wet ground through P150, P320, P800 and P2000 silicon carbide abrasive papers and polished using liquid SiO_2_ (~50 nm in diameter) suspension solution until the ‘mirror-like’ surface finish. In addition, a transmission electron microscope (TEM) specimen of Zircaloy-4 was prepared by a standard preparation technique, which includes cutting of a disk 3 mm in diameter, grinding, and electro-polishing in a twin-jet electropolisher with a chemical solution of 10 vol.% perchloric acid (HClO_4_) and 90 vol.% ethanol (C_2_H_5_OH) at −30 °C until perforation occurred.

### Electron beam irradiation

Electron beam irradiation was conducted at room temperature in a FEI Inspect F50 field-emission scanning electron microscope (FE-SEM) under high vacuum using electron beam at 30 kV accelerating voltage at 204 μA emission current. During irradiation, the FE-SEM was operated in line scan mode at 20,000× magnification with a selected area of ~14.9 × 12.8 μm^2^ focused by electron beam. The electron irradiation dosage was controlled by the number of sustained electron beam scans (every scan lasts 35 s to obtain a SEM image), that is, the irradiation time, as the specimen current density was constant during irradiation. 32 sustained electron beam scans were performed on the polished surfaces of bulk pure Zr and Zircaloy-4 specimens, respectively. In addition, a thin film region of the TEM specimen of Zircaloy-4 was irradiated for 32 sustained electron beam scans in the FE-SEM and subsequently observed in a JEM 2100 F TEM. Such alternative treatments of electron beam irradiation in the FE-SEM and microstructure observation in the TEM were repeated for 4 times (TEM observations were carried out after irradiation for 32, 64, 96, and 128 scans, respectively).

### Characterization

Morphology observations during electron beam irradiation were performed in the FEI Inspect F50 FE-SEM under high vacuum at 30 kV electron accelerating voltage at 204 μA electron emission current. During FE-SEM observations, chemical compositions were analyzed with energy dispersive X-ray spectrometer (EDS) attached to the FE-SEM. TEM observation was conducted in a JEM 2100 F TEM under high vacuum operating at 200 kV electron accelerating voltage at 204 μA electron emission current at ~40 pA/cm^2^ specimen current density. During TEM observations, bright field (BF) TEM images, selected area electron diffraction (SAED) patterns, high-resolution transmission electron microscope (HRTEM) images, scanning transmission electron microscope (STEM) images, and elemental maps were taken for analysis. In order to analyze HRTEM images, Digital Micrograph (Gatan Inc.) software was used to obtain fast Fourier transformation (FFT) diffraction patterns and inverse fast Fourier transformation (IFFT) images.

## Electronic supplementary material

Video Legend

Considerable knock-on displacement of zirconium atoms under a low energy electron beam
