# Supplementary material for: Considerable knock-on displacement of metal atoms under a low energy electron beam
Source: Sci Rep. 2017 Mar 15;7:184. doi: 10.1038/s41598-017-00251-3 (PMC5428036; doi:10.1038/s41598-017-00251-3)
Supplement: Supplementary file 1 — Video Legend [file 41598_2017_251_MOESM1_ESM.pdf]

# Considerable knock-on displacement of metal atoms under a low energy electron beam

Hengfei Gu [a,b], Geping Li [a,c*] Chengze Liu [a,c], Fusen Yuan [a,c], Fuzhou Han [a,c], Lifeng Zhang [a,b], Songquan Wu [d]

[a] *Institute of Metal Research, Chinese Academy of Sciences, 72 Wenhua Road, Shenyang 110016, People's Republic of China*

[b] *University of Chinese Academy of Sciences,19 Yuquan Road, Beijing 100049, People's Republic of China*

[c] *University of Science and Technology of China, 96 Jinzhai Road, Hefei 230026, People's Republic of China*

[d] *Key Laboratory of Optoelectronic Materials Chemical and Physics, Fujian Institute of Research on The Structure of Matter, Chinese Academy of Sciences, 155 Yangqiao Road West, Fuzhou 350002 , People's Republic of China*

## Video Legend

**Considerable knock-on displacement of zirconium atoms under a low energy electron beam.** Under focused and stationary electron beam at 30 kV in a field-emission scanning electron microscope (FE-SEM), nanoscale precipitates within the surface layer of Zircaloy-4 specimen gradually emerged and became clearly visible with increasing the irradiation time.
